# Characterization of the complete chloroplast genome of *Phtheirospermum japonicum* (Rhinantheae)

**DOI:** 10.1080/23802359.2019.1673254

**Published:** 2019-10-09

**Authors:** Yong-Chao Li, Xin-Liang Zhao, Jing Yang, Qing-Yun Fu

**Affiliations:** School of Life Science and Technology, Henan Institute of Science and Technology, Xinxiang, Henan Province, China

**Keywords:** *Phtheirospermum japonicum*, chloroplast genome, phylogenetic analysis

## Abstract

In this study, we report the complete chloroplast (cp) genome of *Phtheirospermum japonicum* was determined through Illumina sequencing method. The complete chloroplast genome of *Ph. japonicum* was 153,397 bp in length and contained a pair of IR regions (25,601 bp) separated by a small single copy region (17,728 bp) and a large single copy region (84,467 bp). The cp genome of *Ph. japonicum* encoded 125 genes including 86 protein-coding genes, 31 tRNA genes and eight ribosomal RNA genes. The overall GC content of *Ph. japonicum* cp genome is 38.3%. By phylogenetic analysis using ML method, *Ph. japonicum* was placed in family Orobanchaceae and showed the closest relationship with *Castilleja paramensis*.

*Phtheirospermum japonicum,* belonging to Rhinantheae (Orobanchaceae) is a hemiparasitic plant that widely distributed in Eastern Asia. In this study, we report the chloroplast genome of *Ph. japonicum* using next-generation sequencing, aiming to figure out its phylogenetic position precisely through molecular phylogenic analysis.

Plant materials of *Phtheirospermum japonicum* sequenced in this study were collected from hengduan mountains of Southwest China (27°30'N, 99°61'E). Both plant materials and total genomic DNA that extacted from fresh young leaves using cetyltrimethylammonium bromide (CTAB) method was stored in Henan Institute of Science and Technology. The corresponding specimen was also stored in herbarium of Henan Institute of Science and Technology under accsion NO. IST-20190611-Phjap.

For high-throughout sequencing (NGS), paired-end library from DNA extracts were prepared with a NEBNext Library building kits, following manufacturer’ s protocol. Then, the library was sequenced on an Illumina HiSeq2500 platform. After reads quiality filtration, the clean reads were assembled by SPAdes 3.11.0 (Bankevich et al. 2012). We used chloroplast genome of *Schwalbea americana* (accession NO.: HG738866) as a reference sequence to align the contigs and identify gaps. To fill the gap, Price (Ruby et al. 2013) and MITObim v1.8 (Hahn et al. 2013) were applied and Bandage (Wick Ryan et al. 2015) was used to identify the borders of the IR, LSC, and SSC regions. The complete sequence was primarily annotated by Plann (Huang et al. 2015) combined with manual correction. All tRNAs were confirmed using the tRNAscan-SE search server (Lowe and Eddy 1997). Other protein-coding genes were verified by BLAST search on the NCBI website (http://blast.ncbi.nlm.nih.gov/), and manual correction for start and stop codons were conducted. This complete chloroplast genome sequence together with gene annotations was submitted to GenBank under the accession numbers of MN075943.

The chloroplast genome of *Ph. japonicum* is a typical quadripartite structure with a length of 153,397 bp. The whole cp genome contains a large single-copy (LSC) region of 84,467 bp, a small single-copy (SSC) region of 17,728 bp, and two inverted repeat (IRs) regions of 25,601 bp. The cp genome possesses 125 genes, including 86 protein-coding genes, 8 ribosomal RNA genes (4 rRNA species) and 31 tRNA genes. The overall GC content of the cp genome is 38.1%.

To infer the phylogenetic relationships between *Ph. japonicum* and the related species, the whole cp genome sequences of 11 species of Lamiales were aligned using HomBlocks (Guiqi et al. 2017), resulting in 101,544 positions in total. The whole genome alignment was analyzed by IQ-TREE version 1.6.6 (Nguyen et al. 2015) under the GTR + F+R2 model. The tree topology was verified under both 1000 bootstrap and 1000 replicates of SH-aLRT test. As shown in Figure 1, the phylogenetic positions of these 11 cp genomes were successfully resolved with full bootstrap supports across almost all nodes. *Phtheirospermum japonicum* was placed in family Orobanchaceae and showed the closest relationship with *Castilleja paramensis*.

**Figure 1.**
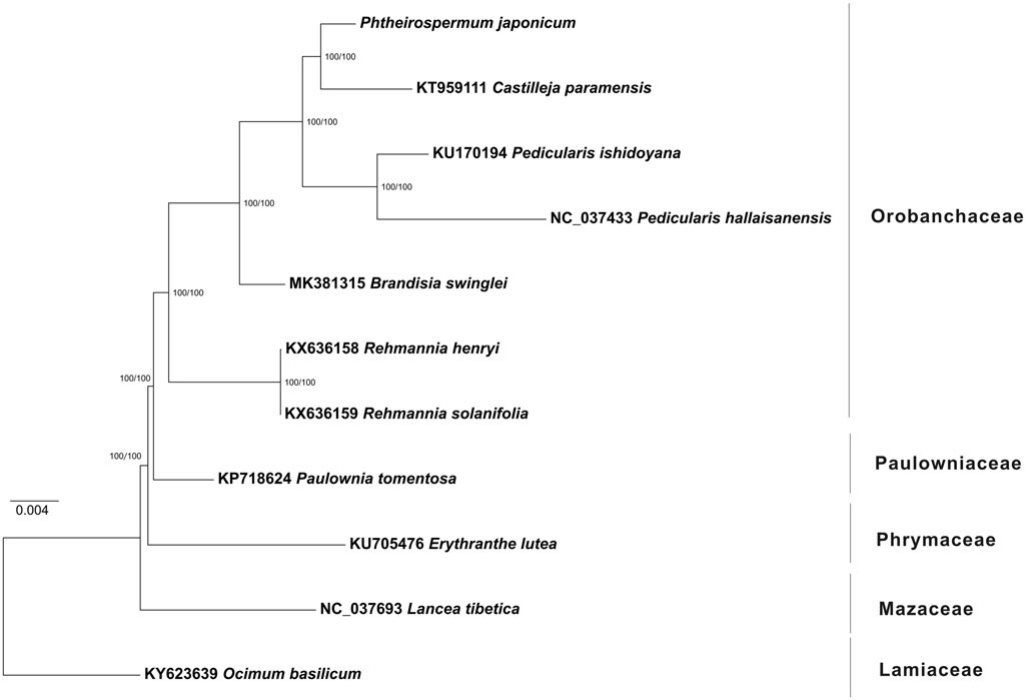
Phylogenetic tree yielded by ML analysis of 11 lamiids cp genomes. Nodes harvesting both full bootstrap and SH-like aLRT values are indicated by pink points. Scale indicates substitutions per site.
